# The Mediterranean Diet and Cognitive Function among Healthy Older Adults in a 6-Month Randomised Controlled Trial: The MedLey Study

**DOI:** 10.3390/nu8090579

**Published:** 2016-09-20

**Authors:** Alissa Knight, Janet Bryan, Carlene Wilson, Jonathan M. Hodgson, Courtney R. Davis, Karen J. Murphy

**Affiliations:** 1 School of Psychology, Social Work and Social Policy, University of South Australia, Adelaide 5001, Australia; janet.bryan@unisa.edu.au; 2 Alliance for Research in Exercise, Nutrition and Activity (ARENA), School of Health Sciences, University of South Australia, Adelaide 5001, Australia; courtney.davis@mymail.unisa.edu.au (C.R.D.); karen.murphy@unisa.edu.au (K.J.M.); 3 Flinders Centre for Innovation in Cancer, School of Medicine, The Flinders University of South Australia, G.P.O. Box 2100, Adelaide 5001, Australia; carlene.wilson@flinders.edu.au; 4 School of Medicine and Pharmacology, The University of Western Australia, Perth 6000, Australia; jonathan.hodgson@uwa.edu.au

**Keywords:** Mediterranean diet, cognitive function, randomised controlled trial, older adults

## Abstract

Evidence from a limited number of randomised controlled intervention trials (RCTs) have shown that a Mediterranean dietary pattern may reduce the risk of cognitive decline and enhance cognitive function among healthy older adults. However, there are currently no data in non-Mediterranean older adult populations. The present study aimed to address this gap by examining the effect of a Mediterranean dietary pattern (MedDiet) for six months on aspects of cognitive function in a randomised controlled intervention trial (the MedLey study) that extended for a duration of 18 months. In the final analysed cohort, a total of 137 men and women (mean age of 72.1 ± 5.0 years) randomly assigned to either a MedDiet or control diet (HabDiet) (i.e., habitual dietary intake), were assessed on a comprehensive neuropsychological test battery, including 11 individual tests. In multivariable-adjusted models, the MedDiet group did not perform significantly better than the HabDiet control group for executive functioning (adjusted mean differences: +2.53, 95% CI −2.59 to 7.65, *p* = 0.33); speed of processing (adjusted mean differences: +3.24, 95% CI −1.21 to 7.70, *p* = 0.15); memory (adjusted mean differences: +2.00, 95% CI −3.88 to 7.88, *p* = 0.50); visual-spatial ability (adjusted mean differences: +0.21, 95% CI −0.38 to 0.81, 0.48); and overall age-related cognitive performance (adjusted mean differences: +7.99, 95% CI −4.00 to 19.9, *p* = 0.19). In conclusion, this study did not find evidence of a beneficial effect of a MedDiet intervention on cognitive function among healthy older adults.

## 1. Introduction

At present, it is thought that around 47.5 million people worldwide are living with dementia [1]. With extremely limited pharmacological treatment options currently available, and no preventative options for normal cognitive ageing or MCI, this incidence rate is estimated to almost triple by 2050 [2]. The burden of such trajectory will place ever-increasing financial and social demands on the health care system in the future, with extra residential aged-care beds, medical services and health-care providers needed to meet such dependency.

One avenue for slowing the cognitive decline associated with normal aging is optimising dietary behaviour. In particular, increasing scientific and public recognition has been given to the traditional Mediterranean diet (MedDiet) from Crete, Greece. The MedDiet has been highlighted in empirical literature as a potential means to preserve optimal cognitive function in older age and also as a means to prevent and delay the progression from healthy cognitive function to pathological neurodegeneration (i.e., MCI, AD) [3]. It is characterised by high consumption of virgin olive oil, green leafy vegetables, fish, walnuts and seeds, moderate red wine, low consumption of red meat, dairy products and processed foods, and high levels of monounsaturated fatty acids, flavonoids, antioxidants, vitamins E, B_6_, B_12,_, folate, carotenoids, omega-3 and omega-6 polyunsaturated fatty acids [4]. These foods and bionutrients individually have been shown to exhibit neuroprotective properties in the ageing brain and benefit cognitive function [5,6]. The synergistic effects of these elements together in one whole dietary pattern, such as the MedDiet, are less well known [7]. A number of recent cross-sectional and prospective studies have attempted to gain further insight into the relationship between adherence to the overall MedDiet pattern and age-related cognitive function [8,9,10,11,12,13,14,15,16]. The findings from this body of research are promising, however, the results are inconsistent, with some studies showing a significant association between the MedDiet and age-related cognitive function [8,9,13,15], and others revealing no link [10,11,16].

To date, only two known randomised controlled trails (RCTs), both emerging from the overarching principle PREDIMED (PREvención con DIeta MEDiterránea) study, have investigated the MedDiet-cognitive ageing association. In a parallel-group, cardiovascular prevention trial, Martínez-Lapiscina and colleagues [17] investigated the effects of two MedDiet type interventions (i.e., a diet supplemented with extra virgin olive oil (EVOO) or one with mixed nuts) in comparison with a low-fat control diet on cognitive performance among 522 Spanish elderly participants at high cardiovascular risk. Results from this trial, as indicated by adjusted means and 95% confidence intervals (CIs), revealed cognitive performance scores on two global, dementia screening tests, including the Mini-Mental State Examination (MMSE) [18], and a Spanish version of the Clock Drawing Test (CDT) [19] were significantly higher for participants in the MedDiet groups supplemented with either EVOO or mixed nuts, compared with the low-fat control group.

Similar results were documented by Valls-Pedret and colleagues [20], who conducted another parallel-group RCT in Barcelona, Spain with 447 older men and women at high cardiovascular risk (i.e., presence of type 2 diabetes mellitus or at least three of five cardiovascular risk factors: Hypertension, dyslipidemia, smoking, overweight or obesity, and family history of early-onset coronary heart disease). Results from multivariate analyses (mean *z*-scores with 95% CIs) indicated that after confounders were accounted for, participants allocated to the Mediterranean diet plus olive oil group scored significantly higher on the Rey Auditory Verbal Learning Test (RAVLT) [21], a measure of episodic declarative memory compared with the control group (*p* = 0.05). No other statistically significant differences in any of the other cognitive tests were observed between groups. In relation to composite scores, the MedDiet + nuts group improved significantly on the memory composite score compared with the control group in changes from baseline across treatment arms (0.09, −0.05 to 0.23; *p* = 0.04 vs. controls), and the MedDiet + EVOO group improved significantly in the frontal (0.23, 0.03 to 0.43; *p* = 0.003 vs. controls) and global cognition (0.05, −0.11 to 0.21; *p* = 0.005 vs. controls) composite scores over time in comparison to the control group.

While results found in these RCTs indicate that a MedDiet + EVOO and MedDiet + nuts intervention may benefit age-related global cognitive function among older adults at high vascular risk, it remains unclear whether a typical MedDiet pattern without additional supplementation of polyphenol rich olive oil and nuts, offers the same benefit. Furthermore, no clinical trial to date has been conducted exclusively on healthy older adults, without the presence of clinical pathology. There is also extremely scarce evidence documented for the effects of a MedDiet intervention on various cognitive functions and domains other than global cognition, using a comprehensive neuropsychological test battery.

The primary aim of the current study, was to address these gaps by comprehensively examining the effects of a MedDiet pattern versus a habitual dietary pattern on various neuropsychological tests as well as five composite variables including: Overall age-related cognitive function, speed of processing, memory, executive functioning, visual spatial ability among healthy older adult participants (≥65 years) in a six month randomised intervention trial, using a wide-ranging test battery that was selected based on sensitivity to dietary change over six months. It was hypothesized that participants allocated to the MedDiet intervention group would perform significantly better than the control group at the final assessment time point (six months) on a series of cognitive tests measuring aspects of speed of processing, memory, executive functioning, visual–spatial ability, and total age-related cognitive function.

## 2. Methods

### 2.1. Study Design

The MedLey study design was a randomised, controlled, 2-cohort parallel group comparison intervention trial conducted in Adelaide, Australia from August 2013 to February 2015. Using repeated measures every three months, two diets (1. experimental; MedDiet pattern; 2. control; habitual pattern) were assessed for their effects on cognitive performance among a healthy, older adult population. A description of the study protocol, including the study design, procedure and safety considerations have been described in detail elsewhere [22,23].

**Figure 1:**
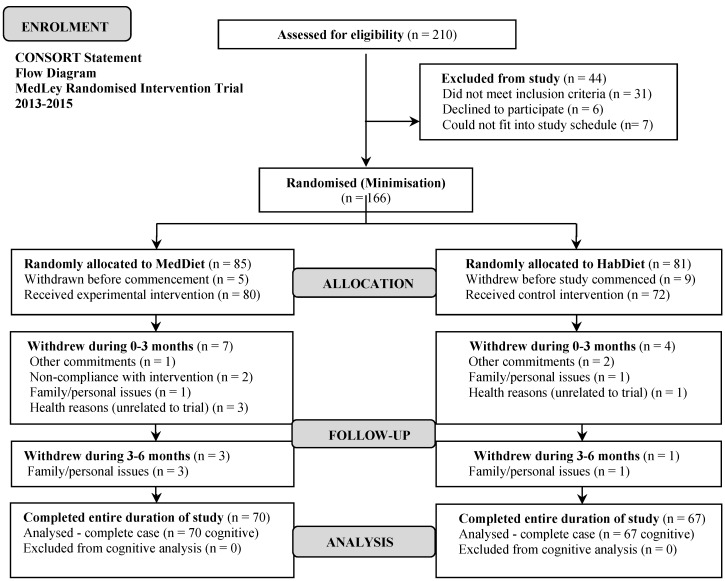
Consolidated Standards of Reporting Trials (CONSORT) flow chart from screening to completion [22].

In brief, a rolling recruitment generated an initial sample of 166 elderly South Australian men and women aged 65 years and above, with normal cognitive function and proficiency in English language. At the initial screening visit, each volunteer went through a detailed medical and neuropsychological history, along with having their height, weight and blood pressure measured, as well as providing a fasting blood sample for the examination of blood lipids, glucose and liver function. Additionally, each volunteer was screened for MCI and dementia using a psychometrically validated screening instrument; the DemTect [24]. Volunteers who had cognitive impairment or dementia (scores of <13 using the DemTect), or who exhibited other potential underlying health issues were excluded from the study and referred to their general practitioner for follow-up. Thus, from an initial 210 volunteers assessed for eligibility, 44 were excluded for reasons aforementioned, leaving 166 eligible participants to be randomised. An additional 14 participants withdrew before the intervention commenced. Eligible participants were assessed at three time points (baseline, 3 months and 6 months) at the Sansom Institute for Health Research, University of South Australia for the evaluation of age-related cognitive function and cardiometabolic outcomes. Across the duration of the intervention, three further participants withdrew as they found difficulty maintaining compliance with the study intervention, three due to other commitments, six due to personal/family issues, and four due to health reasons unrelated to the trial, leaving a total of 137 participants who completed the entire trial, and could be analyzed (see Figure 1).

The Human Research Ethics Committee of the University of South Australia, and Flinders University approved the study (ID: 31163). The trial was registered in the Australian New Zealand Clinical Trials Registry (ACTRN12613000636752), and all participants provided written, informed consent.

### 2.2. Study Population

At baseline, the study population consisted of 166 free-living Australian men and woman aged 65 years and above, who were free of dementia (i.e., obtained a DemTect score ≥13), had healthy age-related cognitive function, and were proficient in English language. In addition, participants had no previous or current conditions that may cause any kind of cognitive impairment, such as, a traumatic head/brain injury, stroke, suffer a neurological or psychiatric condition, or were using medications known to influence cognition. They were free of cardiovascular disease or angina, uncontrolled hypertension (>170/100), malignancy, any major liver, kidney, respiratory or gastrointestinal disease, and had a weight less than 135 kg. Participants were not actively undertaking a weight loss program, smoking, or using any form of appetite suppressant or Orlistat (Xenical), and they were not participating in any another dietary intervention study.

### 2.3. Minimised Randomisation

Following completion of the screening process, eligible participants were de-identified and randomly allocated to one of two dietary groups: Group 1. experimental; Mediterranean Diet (*n* = 85); Group 2. control; Habitual Diet (*n* = 81) through a process of minimisation, whereby participants were matched on age, gender and body mass index (BMI) using predictive methods of allocating participants by knowing factor levels of the previous enrolled participant, and then having the properties of the next participant. Minimised randomisation was chosen as an alternative method to pure randomisation (i.e., participants are randomly allocated into trial groups prior to the commencement of the trial, through means of chance only) in the MedLey trial as a way to overcome the issue of unmatched trail groups. Minimisation aims to allocate participants in such a way that minimises differences among groups, with respect to predictive factors. Thus, we chose this process, along with some elements of pure randomness (i.e., blocking) into the minimisation algorithm, as a way to make the prediction unlikely and overcome the main shortcoming of minimisation; potential invalidation of trial blindness and introduction of selection biases.

The person in charge of the minimised randomisation process was a chief investigator, who was not involved with study participants during any stage of data collection or data analysis. The researcher in charge of administering, assessing and scoring cognitive outcomes was also blind to group assignment.

### 2.4. Power Calculations

Power calculations for the study sample size were determined by examination of effects in previous research [25], and estimated using standard conventions for a study with two equal groups (Zα of 1.96 for 5% level of significance, Z1-β (1-β% power) with β% of type II error (0.84 at 80% power), *r* = n1/n2 ratio for equal sample size for 2 groups and σ and d (the pooled standard deviation and difference of means of 2 groups)). From these calculations, it was estimated that a total sample size of 128 subjects, with two groups of (*n* = 64) would provide 80% power to detect a significant effect size of 0.5 (predicted change/SD of change, *p* < 0.05) [22]. Based from these foundations, it was assumed that any found difference in change in cognitive outcomes would represent a theoretical and clinically meaningful outcome. An initial sample of 166 (an additional 38 volunteers, 19 per group) was targeted to allow for up to a 30% attrition.

### 2.5. Diet Intervention—Mediterranean Diet

A full description of the experimental Mediterranean diet intervention with food groups, condiments, nutrient profile, servings, quantities, and energy from food percentages used in the MedLey study has been described in detail elsewhere [23]. Briefly, at the commencement of the study participants met with a qualified dietitian and were informed which dietary condition they were randomly allocated to (i.e., MedDiet; experimental or HabDiet; control). Thereafter, all participants were closely monitored on a fortnightly basis in an informed meeting with a dietitian to check that the diet was being followed according to compliance standards.

The Mediterranean-style dietary pattern used for the MedLey RCT was based around an in depth literature review of principal studies of the Cretan Mediterranean diet, including Trichopoulou and colleagues [26] and two renowned MedDiet food pyramids [4,27], the Australian Nutrient Reference Values, and Australian standard serving size recommendations. In light of these sources, an Australianised Mediterranean food and nutrient profile was established. A summary of the main food groups adopted for the MedLey RCT is as follows: 1. extra virgin olive oil (EVOO); 2. breads and cereals; 3. legumes; 4. vegetables; 5. fish; 6. fruit; 7. cheese; 8. red wine (upon participants choice, not compulsory); 9. Greek yoghurt; 10. Nuts; 11. potato (white); 12. Milk; and 13. eggs. Free food in the form of legumes, yoghurt (natural/flavoured Greek), Australian EVOO (Cobram Estate), canned tuna (Simplot), walnuts, peanuts and almonds (donated by Almond Board of Australia and peanuts donated by the Peanut Company of Australia) were provided continually throughout the trial to those participants allocated to the MedDiet group as an incentive to improve compliance with the diet. Participants allocated to the control group (HabDiet) were asked to simply maintain their customary lifestyle and dietary pattern. As an incentive to increase study compliance, participants in the control group were provided with monetary gift vouchers to local food supermarkets throughout the trial.

### 2.6. Compliance

Compliance for the present study was achieved via the use of a comprehensive range of compliance measures including: 1. erythrocyte fatty acid composition (marker of MUFA/SFA ratio); 2. plasma carotenoids (CRTs) via blood samples (biomarkers of fruit and vegetable intake); 3. urinary metabolites (potassium, sodium, magnesium and calcium); 4. a semi-quantitative daily food check list; 5. the food frequency questionnaire (FFQ; Cancer Council Victoria); and 6. 3-day weighed food record [WFR]). Collectively, the compliance achieved in the MedLey study was 92%.

### 2.7. Covariate Assessment

A detailed demographic questionnaire was filled out by all participants at baseline containing information about their age, gender, self-rated physical, mental and social health status, highest level of education achieved, marital status, country of birth, parents’ country of birth, income level, current occupation and level of work, perceived adequacy with current income level. In addition, body mass index (BMI), and information relating to family history of diagnosed chronic health conditions and diseases, including cardiovascular and cerebrovascular diseases, diabetes, dementia, and any current medications and vitamin supplements they were gathered to account for potential vascular risk factors. Apolipoprotein E (*ApoE*) genotype was considered dichotomously: presence of at least one ε4 allele (i.e., sum of ε4/4 and ε4/3 geneotypes) versus absence of any ε4 allele. State and trait anxiety symptomatology was assessed on the Spielberger State-Trait Anxiety Inventory Form Y (STAI-Y) [28], and depressive symptomology was assessed on the Centre for Epidemiological Studies Depression Scale (CES-D) [29]. The Perceived Stress Scale (PSS) [30] was used to measure subjective stress levels, a modified version of the Leeds Sleep Evaluation Questionnaire (LSEQ) [31] was used to assess the quality of participants previous night’s sleep, and the Short Form Health Survey (SF-36) was used to evaluate participants subjective health status. These factors were considered potential correlates of the dependent variable (i.e., confounding variables within the context of the current study).

### 2.8. Primary Outcome—Cognitive Assessment

In accordance with prior research [32,33] for the present study, age-related cognitive function was operationalised as “non-pathological changes in aspects of fluid cognition (e.g., speed of processing, memory, executive functioning, attention, visuospatial and visuomotor ability) that occur as a result of normal human ageing”. A full description of the neuropsychological test battery, including the development, rationale and psychometric properties of the chosen tests used in the MedLey study can be found elsewhere [22]. Briefly, a comprehensive battery of eleven cognitive tests were utilised as an overall indication of cognitive function. A principal components factor analysis with oblique rotation was conducted to determine domain specific cognitive factors. Using the criteria of eigenvalues >1, results of the factor analysis revealed four primary factors: 1. executive function, computed as the mean of summed *Z*-scores of the following four tests: Dodrill’s [34] version of the Stroop Test (interference control), Initial Letter Fluency (ILF) and Excluded Letter Fluency (ELF) (strategic retrieval search) and D-KEFS [35] version of the Tower of London (TOL) (planning); 2. memory, computed as the mean of *Z*-scores of the following four tests: Rey and Schmidt’s [21] Rey Auditory Verbal Learning Test (RAVLT) (episodic declarative memory), Digit Span Forward (DSF) (short term memory), Digit Span Backward (DSB) and the Letter-Number sequencing (LNS) (working memory) subtest tasks from the Wechsler Adult Intelligence Scale (WAIS-IV) [36]; 3. speed of processing, computed as the mean of summed *Z*-scores of the following two tests: Symbol Search and Coding core subtests from the WAIS IV [36]; and 4. visual-spatial memory ability, computed as the *Z*-score of the following test: The Benton Visual Retention Test (BVRT) [37]. We also calculated total cognitive function, computed as the mean of summed *Z*-scores of all eleven included individual cognitive tests in the battery.

### 2.9. Statistical Analyses

The data were analysed using SPSS Version 21.0 for Windows (Chicago, IL, USA), with an alpha set at *p* < 0.05. With respect to baseline characteristics of the two diet groups, descriptive statistics were performed for categorical variables (frequencies and percentages) using Pearson's chi**-**squared test (*χ*^2^), and for continuous variables (mean, standard deviation (SD) and the standard error of the mean (SEM)) using one-way analysis of variance (ANOVA) on an intention-to-treat basis. Variables were screened for normality through visual inspections of the distribution using histograms and box plots, along with skewness and kurtosis statistics provided by SPSS. Identified univariate outliers (i.e., 3.29 SD away from the mean) were transformed using the truncation method [38], whereby extreme scores were re-coded to one unit larger or smaller than the next most extreme score in the distribution. However, it should be noted that truncation was only attempted once for each outlier, and not repeated several times until normality was reached. In addition, missing data for continuous variables were imputed using the conservative method of mean substitution. These methods were deemed most appropriate to obtain a normal distribution of the cognitive data as there were no missing data for baseline or the final assessment time point, and only twelve values missing for the three month assessments [39]. As a result, a normally distributed cognitive data set including 137 cases was obtained for subsequent analyses of the primary hypothesis.

Multivariate adjusted mean score differences among the groups were conducted using Mixed Factorial Repeated Measures Analysis of Covariance (ANCOVA) with simple main effects contrasts (EMMEANS). Three time points described the three levels of the within subject factor “Time” (i.e., baseline, 3 months and 6 months). The between subject factor was “Treatment Group” (i.e., MedDiet vs. HabDiet). An interaction effect was hypothesized whereby those in consuming the MedDiet were hypothesized to score better on all cognition factors at 3 months and 6 months than those consuming the HabDiet. Corresponding effect sizes in the form of Partial Eta^2^, 95% confidence intervals, and Power statistics (i.e., Power = 1-Beta; function of effect size and sample size, probability of not making a type 2 error) were calculated for all hypothesized associations to provide an evaluation of the magnitude of effect (i.e., practical significance).

A correlation matrix was conducted using SPSS Predictive Analytics Software (version 21.0, IBM Corporation, New York, NY, USA), which identified correlations between all potential covariates, including: age, sex, BMI, education, presence of Apolipoprotein E-ε*4* allele, self-rated depressive symptoms, sleep quality, anxiety symptoms and stress levels, family history of dementia, AD, incidence of heart attack, high blood pressure, stroke and diabetes, physical activity and the dependent variables (i.e., all individual cognitive test scores, and composite scores for: overall age-related cognitive performance, executive function, speed of processing, memory, visual-spatial ability. Only variables with significant correlations (*p* < 0.05) were thereafter included in the main statistical analyses [40]. These were age, sex and depression. Finally, post hoc analyses investigated the association between ApoE genotype and group allocation and cognition.

## 3. Results

### 3.1. Descriptive Statistics

#### 3.1.1. Participant Characteristics

The analysed cohort consisted of 137 included participants (*n* = 64 male, *n* = 73 women) for the present analysis. Descriptive results generated from an “intention-to-treat” basis showed that at baseline the mean age (±SD) of participants was 72.0 ± 4.94 and BMI was 26.7 ± 3.79. In relation to ApoE genotypes, 24.8% had the presence of at least one E4 allele, while 38.1% had no E4 allele. Most participants (50.7%) were born in Australia, 38.4% of participants reported their birthplace as Europe, 2.9%, Asia, and 1.4% North America. The majority of participants were married (64.5%), with 15.2% divorced, 8.0% single, and 2.2% de facto. The highest level of education obtained was mostly secondary schooling (40.6%), followed by TAFE (21.0%), a university undergraduate degree (18.8%), a university postgraduate degree (10.1%), and a doctorate (2.2%). The vast majority of participants were retired at study commencement (76.1%), with 9.4% working full-time, and 1.4% working part-time. Finally, 50.0% considered their subjective overall health status to be very good, 37.7% considered it to be good, while 4.3% considered it to be satisfactory. Table 1 shows the full baseline characteristics of participants by intervention group.

#### 3.1.2. Changes in Diet Observed in Experimental and Control Groups

Descriptive statistics were performed to evaluate the observed changes in nutrient and food intake between baseline and the final assessment point for both the experimental MedDiet group and the control HabDiet group. Table 2 shows a number of significant changes (*p* < 0.05) in various nutrients among the MedDiet group. Specifically, the greatest of those changes were observed for: Cholesterol, sugars, Vitamin E, Zinc, MUFA:SFA, total long-chain n-3, linoleic acid, fat as MUFA, fat at SFA, kJ from fat, kJ from SFA, kJ from MUFA, kJ from carbohydrate. All of these changes were in the predicted direction aside from Zinc intake. In contrast, as expected, less dietary changes between baseline and the final assessment time point were observed for the control HabDiet group. Potassium intake, as well as a-linolenic acid intake, significantly decreased, and somewhat unexpectedly, sugar intake decreased and fat as MUFA increased over the duration of the trial. Thus, both diets (MedDiet and HabDiet) changed over the course of the trial in such a way that tended to incorporate higher levels of bioactive nutrients at the end of the trial period compared to baseline, and lower levels of various nutrients known to exacerbate age-related cognitive function (e.g., sugars and sodium).

Table 3 shows that participants in the MedDiet group significantly increased their consumption of extra virgin olive oil, fruits, legumes, nuts, dairy, fish and seafood, and significantly decreased their intake of small goods, discretionary foods and miscellaneous foods. There were no significant change in vegetables (including potatoes), breads and cereals, eggs, red wine, discretionary beverages and coffee. Within the HabDiet group, fish and seafood, red wine and miscellaneous foods were significantly lower at the final assessment point. No significant changes in the other food groups were observed.

### 3.2. Inferential Statistics

#### Primary Outcome: Age-Related Cognitive Function

A Mixed Factorial Repeated Measures ANCOVA analysis with simple main effects contrasts (EMMEANS) were conducted to evaluate the effect of a MedDiet intervention group versus a control habitual diet group on cognitive performance outcomes, comparing baseline assessment scores with 6 months assessment scores. The multivariable adjusted mean results presented in Table 4 shows that after covariates were accounted for, there were no significant between-groups mean differences in performance between participants allocated to the experimental MedDiet group and those allocated to the HabDiet control group on cognitive outcomes including: executive functioning (adjusted mean differences: +2.53, 95% CI −2.59 to 7.65, *p* = 0.33); speed of processing (adjusted mean differences: +3.24, 95% CI −1.21 to 7.70, *p* = 0.15); memory (adjusted mean differences: +2.00, 95% CI −3.88 to 7.88, *p* = 0.50); visual-spatial ability (adjusted mean differences: +0.21, 95% CI −0.38 to 0.81, 0.48); and overall age-related cognitive performance (adjusted mean differences: +7.99, 95% CI −4.00 to 19.9, *p* = 0.19). Table 5 shows that no interaction effects were found for executive functioning, *F*(2, 133) = 2.25, *p* = 0.11, Partial Eta^2^ = 0.03, Power = 0.45; speed of processing, *F*(2, 133) = 0.21, *p* = 0.81, Partial Eta^2^ = 0.03, Power = 0.08; memory, *F*(2, 133) = 0.83, *p* = 0.44, Partial Eta^2^ = 0.01, Power = 0.19; visual–spatial ability, *F*(2, 133) = 0.54, *p* = 0.58, Partial Eta^2^ = 0.01, Power = 0.14; and overall age-related cognitive performance, *F*(2, 133) = 2.35, *p* = 0.09, Partial Eta^2^ = 0.03, Power = 0.46. Essentially, such findings indicate that there were no statistically significant difference between the MedDiet and the HabDiet groups on cognitive outcomes after 6 months follow-up. There were, however, significant within-subjects effects of Time for the BVRT test score, the overall RAVLT score and the memory composite score, however such effects were most likely simply a result of practice effects due to the nature of the study.

## 4. Discussion

To our knowledge, this was the first RCT to evaluate, among an Australian sample of healthy older adults (≥65 years) rather than a Mediterranean sample, the effect of a MedDiet pattern versus a HabDiet (control) pattern on a comprehensive range of cognitive functions. Results of this trial revealed that participants randomly allocated through minimisation to a MedDiet did not perform significantly better on tests of cognitive function than participants in the control HabDiet group after six months intervention. Our findings contrast with the two other known RCTs conducted in the area to date [17,20]. Both of these RCTs found a longitudinal, statistically significant benefit of a MedDiet intervention supplemented with either EVOO or nuts on age-related cognitive function among older adults, independent of cofounders. In relation to cross-sectional and prospective studies in the area of MedDiet-healthy cognitive function, our finding of no effect is consistent with a number of published studies [10,11,16,41] but not all.

While these results were contrary to our expectations, there are several potential explanations for the null findings. The present RCT analysed a sample of 137 participants at the completion of the trial and was conducted over 6 months, while the previous two RCTs [17,20] analysed larger samples (*n* = 522 and *n* = 447) and were conducted over a longer period of time (6.5 years and 4.1 years), respectively. Thus, it is possible that the smaller sample size and shorter study length of the present RCT lacked statistical power to detect an effect of the MedDiet intervention on cognitive function.

Another possible explanation for the present findings may relate to the differing population characteristics of Mediterranean versus non-Mediterranean studies. The mostly Australian-born, non-Mediterranean population of the present study would have had robust differences in socio-cultural values, norms, attitudes, ways of living and dietary habits compared to that of the Spanish, Mediterranean populations used in Martínez-Lapiscina et al.’s [17] and Valls-Pedret et al.’s [20] studies. Thus, it is possible that such innate socio-cultural differences may or may not have contributed to the contrasting findings between the three studies. As the present study is the only non-Mediterranean RCT to investigate the effects of a MedDiet on cognitive function among older adults (≥65 years) that we know of, further investigation will be needed to validate or negate this proposition.

Another possible explanation for the null result is the bioactive content of the diet. The PREDIMED diet compared a MedDiet supplemented with additional EVOO or nuts, providing additional MUFA and polyphenolic compounds from the EVOO and protein and MUFA and alpha linolenic acid from the mixture of hazelnuts, walnuts and almonds. While these two elements are unquestionably key components of the traditional MedDiet pattern, with amplified levels of these in the intervention diet, higher concentrations of specific bioactive nutrients in the brain (e.g., monounsaturated fatty acids, phenolic antioxidants, and polyphenols) may potentially change the normal synergistic chemistry found among the interacting nutrients of a balanced MedDiet pattern, thus, altering the underlying molecular mechanisms. Further empirical discussion is required to determine the effectiveness of individual key nutrients and foods of the MedDiet versus the proposed synergistic effects of the whole MedDiet pattern. It is possible that while the whole MedDiet pattern appears to provide the ultimate balance of bioactive nutrients for longevity and CVD, the real benefit to age-related cognition may come from an exaggerated supplementation of key hypothesized nutrients and foods in isolation.

Another potential explanation may relate to the fact that the present study sample was made up of highly educated people, who were generally very healthy. Over 31.1% of this study sample had graduated with a university degree. Furthermore, 87% of participants subjectively reported they found their health to be either very good or good, indicating that the overall educational level and health of the present sample was potentially above average for an older adult population. Indeed, in line with the cognitive reserve hypothesis [42], there is accumulated evidence that during older age, those who have a higher level of experiential resources (e.g., knowledge, IQ, education, occupational attainment) exhibit higher levels of age-related cognitive function [43]. Furthermore, there is documented evidence that older adults who lead a more active an healthy lifestyle elicit more efficient neuronal networks which act to preserve cognitive function (i.e., the environmental complexity hypothesis) [44]. As such, it is possible that the general level of cognitive reserve and good health practices inherent within both the experimental and control groups in the MedLey study before the study commenced, thwarted any real effects of a six month MedDiet intervention on cognitive function among this study sample.

Along the same line of thought, it has been suggested that the therapeutic benefit of the MedDiet may be more exclusively reserved for preventing the development of pathological cognitive ageing over preserving normal cognitive ageing [9]. Indeed, in a recent review it was found that there was an across-the-board consensus that the MedDiet was positively associated with reduced MCI, AD and dementia among older adults. In contrast, there was a mixed agreement among studies on the benefit of the MedDiet for normal cognitive ageing outcomes [45]. Interestingly, in a large prospective study conducted by Feart and colleagues [9], a positive association between MedDiet adherence and higher MMSE scores (global cognitive function) was found (β = −0.006; 95% (CI), −0.01 to −0.0003; *p* = 0.04) among older individuals free of dementia; a hallmark of pathological cognitive ageing. However, results indicated that the associations between MedDiet adherence and test scores of fluid cognitive abilities (the cognitive abilities most sensitive to normal age-related cognitive change) [45] were not significantly associated, concurring with the findings in the present study. It may be that the greatest window of opportunity for the MedDiet to promote its effects lies, not in the healthy or late clinical stages of age-related cognitive function, but rather in the prodromal phase of AD, often referred to as MCI. The implications of gaining insight to such proposed conjecture would be greatly useful for the area, and should be considered by future research.

This trial has several strengths. First, to our knowledge, the MedLey study was the first non-Mediterranean RCT to investigate the effect of a MedDiet intervention on cognitive function among a non-clinical, healthy older adult sample (≥65 years). Second, the present study was a well-controlled, dietary intervention trial, where participants were monitored meticulously by dietitians through all stages of the intervention to ensure compliance >90% to the dietary intervention was obtained. Third, both the MedDiet and the cognitive test battery chosen for the MedLey study were based upon detailed systematic reviews [46,47] corresponding to a MedDiet and cognitive test battery with notable methodological rigour.

This study also has limitations. The trial was a single-site recruitment study, resulting in a restricted study sample of fairly well-educated, healthy volunteers from metropolitan Adelaide. Generally speaking, these particular characteristics tend to correspond with older adults who actively enroll in the kind of research devoted to generating important public health knowledge for the community. Thus, the study sample may not have represented the wider older adult population in Australia with lower levels of education and poorer health, and from different geographical locations. As such, the generalisability of the study findings to the average older adult population is uncertain. Additionally, the study length of the MedLey trail (6 months) along with the sample size (*n* = 137) may not have provided adequate statistical power to establish any real intervention effect. Finally, the present study was single blinded. However, for randomised trials in the area of nutrition whereby explicit dietary guidance and allocation of foods is given to participants on a regular basis, conducting a double-blind study is not viable.

## 5. Conclusions

In conclusion, the results of this study have contributed to the body of existing literature by showing that in the first-ever conducted RCT using a healthy non-Mediterranean older adult population 65 years and above, there were no statistical differences in cognitive performance between those randomised to a MedDiet compared with those on a control diet. Exploring the validity of this finding through future studies using larger study samples, longer study durations and extrapolated neuropsychological and dietary assessment methods will be extremely important and relevant for non-Mediterranean countries. This type of collaborative evidence will strengthen the idea of whether or not a MedDiet intervention offers a valid benefit to cognitive function or not, particularly in non-Mediterranean older adult populations.

## Figures and Tables

**Table 1:** Baseline characteristics of participants according to allocated group.

Variables at Baseline	MedDiet (*n* = 70) ^a^	HabDiet (Control) (*n* = 67) ^a^	*p*-Value
Age, Mean (SD) ^c^	72.1 (4.9)	72.0 (5.0)	0.960
Sex (Women, *N*, %) ^a,b^	33 (24.1)	40 (29.2)	0.098
Body Mass Index (kg/m^2^) ^c^	26.5 (3.5)	26.9 (4.1)	0.512
Birth country Australia (*N*, %) ^a,b^	40 (29.2)	30 (21.9)	0.521
Married (*N*, %) ^a,b^	48 (35.0)	50 (36.5)	0.318
Education only secondary (*N*, %) ^a,b^	27 (19.7)	29 (21.2)	0.549
Retired (*N*, %) ^a,b^	63 (46.0)	50 (36.5)	0.237
Risk factors (*N*, %) ^a,b^			
Presence of at least one ApoE-4 genotype	24 (17.5)	30 (21.9)	0.163
Family history of heart attack	27 (19.7)	25 (18.2)	0.845
Family history of stroke	17 (12.4)	19 (13.9)	0.799
Family history of diabetes	20 (14.6)	22 (16.1)	0.688
Family history of high blood pressure	32 (23.4)	23 (16.8)	0.502
Family history of dementia	16 (11.7)	10 (7.3)	0.298
Health and psychological status score, Mean (SD) ^c^			
SF-36 Total Physical Component	51.6 (6.7)	52.0 (6.4)	0.735
Total Mental Component	54.7 (7.4)	55.7 (6.1)	0.379
CES-D	34.3 (3.4)	34.6 (4.2)	0.629
PSS	19.9 (4.2)	18.8 (3.9)	0.108
LSEQ	14.7 (5.6)	15.0 (16.3)	0.727
STAI-Y (state)	47.0 (4.2)	46.4 (4.4)	0.387
STAI-Y (trait)	46.4 (4.2)	45.7 (3.8)	0.376
Cognitive assessment score for individual tests, Mean (SD) ^c^			
Stroop Test (interference score)	2.5 (0.5)	2.5 (0.6)	0.398
DSB	9.2 (2.2)	9.3 (2.1)	0.864
BVRT	6.2 (1.5)	6.1 (1.3)	0.744
TOL	15.4 (3.4)	15.9 (3.3)	0.336
RAVLT (total score)	76.7 (14.9)	75.7 (13.8)	0.689
Symbol Search	19.3 (4.1)	19.5 (4.5)	0.812
DSF	11.2 (2.0)	10.9 (2.1)	0.303
LNS	20.5 (2.5)	20.9 (2.8)	0.376
ILF	24.7 (8.5)	25.4 (8.5)	0.651
ELF	23.3 (9.4)	21.9 (7.8)	0.329
Coding	40.7 (9.3)	42.8 (10.8)	0.240

The MedLey study. Abbreviations: BVRT = The Benton Visual Retention Test; CES-D = Centre for Epidemiological Studies Depression Scale; DemTect; DSB = Digit Span Backward; DSF = Digit Span Forward; ELF = Excluded Letter Fluency; ILF = Initial Letter Fluency; LNS = Letter-Number sequencing; LSEQ = Leeds Sleep Evaluation Questionnaire; TOL = Tower of London; PSS = Perceived Stress scale; RAVLT = Rey Auditory Verbal Learning Test; SD = Standard deviation; SF-36 = Short Form Health Survey; STAI-Y = The Spielberger State-Trait Anxiety Inventory Form Y. ^a^ *N* = number of participants; ^b^ % = *χ*^2^ Test (percentages); ^c^ Mean (SD) = one-way analysis of variance mean score and standard deviation.

**Table 2:** Changes in nutrient intake from baseline to the final assessment point observed for the experimental MedDiet group and control HabDiet group.

	MedDiet (Mean, SD)			HabDiet (Mean, SD)		
Nutrients	Baseline	Final Assessment Point ^a^	*p*-Value for Change	Baseline	Final Assessment Point ^a^	*p*-Value for Change
Energy (kJ)	8954 ± 2190	8827 ± 1987▼	0.59	8809 ± 2011.3	8408 ± 2138.7▼	0.16
kJ from protein (%)	19.0 ± 3.0	19.4 ± 3.1▲	0.43	19.0 ± 3.3	19.2 ± 3.6▲	0.73
kJ from fat (%)	33.6 ± 6.0	38.8 ± 7.4▲	<0.001	34.4 ± 5.8	35.6 ± 5.7▲	0.16
kJ from saturated fat (%)	12.1 ± 2.8	9.1 ± 1.8▼	<0.001	12.8 ± 3.0	13.0 ± 3.3▲	0.63
kJ from monounsaturated fat (%)	13.2 ± 3.6	19.7 ± 4.7▲	<0.001	13.2 ± 3.4	14.2 ± 3.6▲	<0.05
kJ from carbohydrate (%)	42.2 ± 7.0	37.8 ± 6.0▼	<0.001	41.3 ± 6.9	40.7 ± 7.5▼	0.25
kJ from alcohol (%)	2.0 ± 5.6	1.8 ± 4.4▼	<0.05	1.7 ± 5.1	2.6 ± 5.5▲	0.69
Fat as mono (%)	42.5 ± 5.9	54.3 ± 5.2▲	<0.001	41.5 ± 5.8	43.2 ± 6.2▲	<0.03
Fat as saturated (%)	39.7 ± 7.9	25.4 ± 4.0▼	<0.001	40.7 ± 7.5	39.9 ± 8.6▼	0.46
Cholesterol (mg)	304.1 ± 138.6	223.4 ± 90.3▼	<0.001	300.8 ± 128.4	296.3 ± 130.9▼	0.82
Sugars (g)	109.5 ± 36.4	99.1 ± 31.3▼	<0.01	107.8 ± 43.6	98.0 ± 39.5▼	<0.02
MUFA:SFA	1.2 ± 0.5	2.2 ± 0.5▲	<0.001	1.1 ± 0.4	1.2 ± 0.05▲	0.08
Fibre (g)	31.3 ± 12.6	33.8 ± 12.4▲	0.21	28.4 ± 8.1	25.7 ± 8.2▼	<0.01
Vitamin C (mg)	157.1 ± 90.3	166.5 ± 76.7▲	0.43	139.4 ± 69.9	120.7 ± 72.9	0.07
Vitamin E (mg)	11.4 ± 6.5	17.3 ± 5.8▲	<0.001	10.8 ± 4.1	10.9 ± 5.0▲	0.89
Total folate (µg)	493.8 ± 237.0	489.5 ± 154.1▼	0.88	425.3 ± 167.5	388.4 ± 148.4▼	0.12
Total vitamin A equivalents (µg)	1100.0 ± 579.6	921.2 ± 682.2▼	0.24	927.1 ± 621.9	893.9 ± 629.6▼	0.53
β-carotene equivalents (µg)	4418.3 ± 3469.6	4508.6 ± 4493.6▲	0.12	3623.8 ± 3377.9	3370.5 ± 3287.5▼	0.38
Sodium (mg)	2367.3 ± 891.0	1792.7 ± 656.3▼	<0.001	2357.7 ± 874.0	2154.8 ± 648.2▼	0.09
Potassium (mg)	3982.7 ± 1451.9	3863.6 ± 978.8▼	0.47	3661.6 ± 817.4	3333.0 ± 807.3▼	<0.01
Calcium (mg)	978.3 ± 381.2	926.2 ± 279.8▼	0.22	927.8 ± 352.2	869.0 ± 342.5▼	0.07
Iron (mg)	14.5 ± 5.6	13.7 ± 3.7▼	0.20	12.8 ± 3.7	12.2 ± 4.4▼	0.35
Zinc (mg)	12.5 ± 4.4	10.9 ± 3.1▼	<0.02	11.7 ± 3.5	12.1 ± 4.5▲	0.43
Total long-chain n3 (mg)	222.7 ± 601.9	586.8 ± 1201.05▲	<0.001	265.7 ± 447.3	190.9 ± 282.5▼	<0.05
Linoleic acid (g)	11.5 ± 6.7	15.2 ± 6.3▲	<0.001	11.4 ± 4.6	10.8 ± 5.0▼	0.40
α-linolenic acid (ala) (g)	1.5 ± 0.7	1.6 ± 1.4▲	0.39	1.5 ± 0.7	1.3 ± 0.5▼	<0.01

Note: ^a^ ▲ = an observed increase in nutrient intake at the final assessment point compared with baseline. ▼ = an observed decrease in nutrient intake at the final assessment point compared with baseline. Abbreviations: g = grams; kJ = kilojules; MUFA = monounsaturated fatty acid; mg = milligrams; mL = millilitres; PUFA = polyunsaturated fatty acid; SFA = saturated fatty acid; µg = micrograms.

**Table 3:** Changes in food intake from baseline to the final assessment point observed for the experimental MedDiet group and control HabDiet group.

	MedDiet (Mean, SD)			HabDiet (Mean, SD)		
Foods	Baseline	Final Assessment Point ^a^	*p*-Value for Change	Baseline	Final Assessment Point ^a^	*p*-Value for Change
Extra virgin olive oil ^†^	0.0 ± 4.5	36.3 ± 28.2▲	<0.001	0.0 ± 7.7	0.0 ± 6.9▼	0.42
Vegetables ^1^	227.2 ± 191.7	256.9 ± 131.2▲	0.22	202.4 ± 119.7	207.7 ± 119.7▲	0.77
Fruits ^2^	272.8 ± 152.1	381.9 ± 195.3▲	<0.001	272.2 ± 154.6	288.1 ± 160.8▲	0.36
Legumes ^†^	0.0 ± 12.6	36.0 ± 47.3▲	<0.001	0.0 ± 11.3	0.0 ± 26.7▲	0.51
Nuts ^†^	6.2 ± 20.0	34.0 ± 37.875▲	<0.001	12.0 ± 20.3	6.3 ± 25.0▼	0.78
Dairy ^3,†^	210.8 ± 243	310.0 ± 225▲	<0.001	213.5 ± 258.5	219 ± 299.2▲	0.30
Breads and cereals	149.9 ± 84.9	146.8 ± 63.7▼	0.80	148.2 ± 70.6	142.7 ± 74.0	0.60
Fish and seafood ^†^	24.7 ± 69.58	77.0 ± 60.3▲	<0.001	36.7 ± 80.1	28.3 ± 60.0	<0.02
Meat ^4,†^	50 ± 87.7	49.9 ± 72.1▼	0.07	73.0 ± 109.3	80.0 ± 84.3▲	0.27
Smallgoods ^†^	0.0 ± 21.7	0.0 ± 6.8▼	<0.01	0.0 ± 24.3	7.0 ± 28.2▲	0.87
Eggs and egg dishes ^†^	9.2 ± 39.8	16.7 ± 32.7▲	0.38	16.7 ± 41.0	16.3 ± 39.7▼	0.10
Red wine ^†^	0.0 ± 157.6	49.9 ± 198.0▲	0.48	9.9 ± 152.8	0.0 ± 99.0▼	<0.01
Discretionary foods ^5,†^	94.5 ± 92.8	50.0 ± 52.6▼	<0.001	103.2 ± 107.3	79.7 ± 95.85▼	0.23
Discretionary beverages ^6,†^	446.2 ± 533.0	467.3 ± 434.4▲	0.08	483.4 ± 721.5	583.3 ± 589.5▲	0.43
Coffee	319.0 ± 252.8	344.6 ± 247.0▲	0.40	312.9 ± 272.2	320.9 ± 283.4▲	0.67
Miscellaneous ^7,†^	150.0 ± 160.5	23.7 ± 68.0▼	<0.001	139 ± 150.4	80 ± 106▼	<0.001

Note: ^a^ ▲ = an observed increase in nutrient intake at the final assessment point compared with baseline. ▼ = an observed decrease in nutrient intake at the final assessment point compared with baseline. ^†^ Non-normally distributed residuals, median ± interquartile range presented and Wilcoxon Signed Rank test used to determine within-group differences. ^1^ Includes all raw, canned and cooked vegetables and potatoes; ^2^ Includes canned, dried and fresh fruits, excludes juice; ^3^ Milk, cheese, yoghurt and custard included. Excludes cream and ice cream; ^4^ White and red meats, including pork, beef, veal, lamb, mutton, chicken, turkey, duck, kangaroo; ^5^ Includes sugars, confectionary, snack foods, crisps, cakes, pastries, deep fried foods, sweet biscuits, ice cream, cream, and fats other than olive oil; ^6^ Includes milkshakes, alcoholic beverages other than red wine, juices, cordials, soft drink and tea; ^7^ Mixed foods including lasagne and pizza, sauces and condiments, special dietary products, soups.

**Table 4:** Multivariable-adjusted mean differences between the MedDiet intervention group and control group (95% CIs).

	MedDiet (*n* = 70)		HabDiet (*n* = 67)
Outcome	Mean ^a^ (95% CI)	*p*-Value ^a^ (vs. Control)	Mean ^a^ (95% CI)
**Total age-related cognitive function score (composite)**	279.8 (271.5–288.1)		287.8 (279.2–296.4)
Adjusted diff. versus control (95% CI)	+8.00 (−4.00–19.9)	0.19	0 (reference group)
**Executive function score (composite)**	69.6 (66.1–73.2)		72.2 (68.5–75.9)
Adjusted diff. versus control (95% CI)	+2.53 (−2.58–7.65)	0.33	0 (reference group)
**Memory score (composite)**	126.9 (122.9–131.1)		128.9 (124.8–133.2)
Adjusted diff. versus control (95% CI)	+2.00 (−3.88–7.88)	0.50	0 (reference group)
**Speed of processing score (composite)**	77.1 (73.9–80.2)		80.3 (77.1–83.5)
Adjusted diff. versus control (95% CI)	+3.24 (−1.21–7.70)	0.15	0 (reference group)
**Visual–spatial score (composite)**	6.07 (5.66–6.48)		6.29 (5.86–6.71)
Adjusted diff. versus control (95% CI)	+0.21 (−0.38–0.81)	0.48	0 (reference group)

^a^ Mixed-effects factorial repeated measures analysis of covariance, Multivariable estimates of effect (adjusted differences with 95% CI). Covariates included: age, sex and depression. Abbreviations: CI = confidence interval; HabDiet = habitual control diet; MedDiet = Mediterranean diet intervention.

**Table 5:** MedDiet group versus control group on age-related cognitive performance outcomes after 6 month follow-up, interaction effects.

Outcome, Treatment Arm	Mean (95% CI) ^a^		*p*-Value for Mixed Factorial ^a,b^ Repeated Measures ANCOVA			Effect Size (Observed Power) ^a,b^		
	MedDiet Group (*n* = 70)	Control Group (*n* = 67)	Between Groups	Within Groups	Interaction Term	Between Groups	Within Groups	Interaction Term
Total age-related cognitive function score (composite)								
3 months	−0.76 (−2.04 to 0.52)	0.82 (−0.51 to 2.14)	0.34	0.71	0.10	0.01 (0.12)	0.04 (0.52)	0.03 (0.45)
6 months	−0.37 (−1.59 to 0.86)	0.40 (−0.88 to 1.67)						
Executive function score (composite)								
3 months	−0.28 (−0.78 to 0.21)	0.82 (−0.51 to 2.14)	0.44	0.07	0.11	0.01 (0.12)	0.01 (0.10)	0.03 (0.47)
6 months	−0.09 (−0.54 to 0.36)	0.30 (−0.21 to 0.82)						
Memory score (composite)								
3 months	−0.22 (−0.80 to 0.37)	0.23 (−0.38 to 0.84)	0.68	0.05	0.44	0.001 (0.07)	0.05 (0.60)	0.01 (0.19)
6 months	−0.05 (−0.66 to 0.56)	0.05 (−0.58 to 0.68)						
Speed of processing score (composite)								
3 months	−0.20 (−0.58 to 0.19	0.21 (−0.19 to 0.61)	0.17	0.90	0.81	0.01 (0.27)	0.002 (0.07)	0.003 (0.08)
6 months	−0.18 (−0.55 to 0.20)	0.19 (−0.20 to 0.58)						
Visual-spatial score (composite)								
3 months	−0.07 (−0.30 to 0.19)	0.07 (−0.16 to 0.31)	0.34	0.71	0.10	0.002 (0.08)	0.02 (0.32)	0.008 (0.14)
6 months	−0.06 (−0.28 to 0.17)	0.06 (−0.18 to 0.30)						
Stroop Test								
3 months	2.33 (2.20 to 2.45)	2.33 (2.20 to 2.46)	0.72	0.07	0.82	0.001 (0.07)	0.02 (0.32)	0.008 (0.14)
6 months	2.28 (2.20 to 2.39)	2.26 (2.15 to 2.36)						
DSB								
3 months	9.49 (9.05 to 9.93)	9.38 (8.92 to 9.84)	0.95	0.55	0.84	0.001 (0.05)	0.009 (0.15)	0.003 (0.08)
6 months	9.41 (8.87 to 9.95)	9.51 (8.96 to 10.07)						
BVRT								
3 months	5.21 (4.81 to 5.60)	5.45 (5.04 to 5.86)	0.55	0.01	0.55	0.02 (0.39)	0.008 (0.14)	0.009 (0.06)
6 months	6.07 (5.65 to 6.50)	6.29 (5.85 to 6.73)						
TOL score								
3 months	16.20 (15.47 to 16.93)	16.52 (15.76 to 17.28)	0.51	0.81	0.67	0.003 (0.10)	0.003 (0.08)	0.005 (0.11)
6 months	17.49 (16.73 to 18.26)	17.51 (16.72 to 18.30)						
RAVLT score								
3 months	79.70 (76.21 to 83.20)	78.88 (75.26 to 82.51)	0.83	0.03	0.24	0.001 (0.05)	0.05 (0.67)	0.02 (0.31)
6 months	86.18 (82.56 to 89.11)	87.54 (83.78 to 91.31)						
Symbol Search								
3 months	21.39 (20.18 to 22.60)	22.13 (20.88 to 23.38)	0.41	0.31	0.78	0.005 (0.13)	0.02 (0.23)	0.004 (0.09)
6 months	24.83 (23.59 to 26.07)	25.57 (24.28 to 18.30)						
DSF								
3 months	11.18 (10.71 to 11.66)	11.44 (10.95 to 11.94)	0.65	0.10	0.20	0.002 (0.07)	0.03 (0.48)	0.02 (0.34)
6 months	11.04 (10.50 to 11.58)	10.74 (10.18 to 11.30)						
LNS								
3 months	20.40 (19.81 to 20.99)	20.92 (20.30 to 21.53))	0.28	0.570	0.85	0.01 (0.19)	0.005 (0.15)	0.002 (0.08)
6 months	20.68 (19.93 to 21.43)	20.88 (20.09 to 21.67)						
ILF								
3 months	24.37 (22.55 to 26.19)	27.10 (25.21 to 28.99)	0.16	0.45	0.34	0.02 (0.29)	0.006 (0.20)	0.01 (0.29)
6 months	25.60 (23.68 to 27.51)	27.25 (25.27 to 29.24)						
ELF								
3 months	22.39 (20.67 to 24.11))	23.77 (21.99 to 25.56)	0.81	0.50	0.18	0.001 (0.06)	0.007 (0.20)	0.02 (0.47)
6 months	24.26 (22.14 to 26.38)	25.18 (22.97 to 27.38)						
Coding								
3 months	46.07 (43.71 to 48.42)	48.82 (46.40 to 51.25)	0.09	0.59	0.92	0.02 (0.39)	0.008 (0.14)	0.001 (0.06)
6 months	52.34 (49.98 to 54.70)	54.73 (52.30 to 57.16 )						

Abbreviations: ANCOVA = Analysis of Covariance; BVRT = The Benton Visual Retention Test; CI = Confidence interval; DSB = Digit Span Backward; DSF = Digit Span Forward; ELF = Excluded Letter Fluency; ILF = Initial Letter Fluency; LNS = Letter-Number sequencing; TOL = Tower of London; RAVLT = Rey Auditory Verbal Learning Test. ^a^ Mixed-effects factorial repeated measures analysis of covariance, *F* tests used. Covariates included: age, sex and depression; ^b^ Degrees of freedom for between subjects, within subjects and the interaction term are (1, 133), (2, 133), and (2, 133), respectively, In parentheses, the first reported degree of freedom refers to the effect for between groups/times, and the second refers to the error term.
